# Alemtuzumab-induced thyroid eye disease successfully treated with a single low dose of rituximab

**DOI:** 10.1530/ETJ-23-0236

**Published:** 2024-04-11

**Authors:** Ilaria Muller, Sara Maioli, Mirco Armenti, Laura Porcaro, Nicola Currò, Elisabetta Iofrida, Lorenzo Pignataro, Jacopo Manso, Caterina Mian, Jens Geginat, Mario Salvi

**Affiliations:** 1 Department of Clinical Sciences and Community Health, University of Milan, Italy; 2 Endocrinology Unit, Graves’ Orbitopathy Center, Fondazione IRCCS Ca’ Granda Ospedale Maggiore Policlinico, Milan, Italy; 3 Ophthalmology Unit, Fondazione IRCCS Ca’ Granda Ospedale Maggiore Policlinico, Milan, Italy; 4 Department of Specialistic Surgical Sciences, Otolaryngology and Head and Neck Surgery, Fondazione IRCCS Ca’ Granda Ospedale Maggiore Policlinico, Milan, Italy; 5 Endocrinology Unit, Department of Medicine (DIMED), University of Padova, Padova, Italy; 6 National Institute of Molecular Genetics (INGM) “Romeo and Enrica Invernizzi”, Milan, Italy

**Keywords:** thyroid eye disease, Graves’ orbitopathy, immune reconstitution therapy, rituximab, alemtuzumab, lymphocyte depletion

## Abstract

**Introduction:**

Secondary thyroid autoimmunity, especially Graves’ disease (GD), frequently develops in patients with multiple sclerosis (MS) following alemtuzumab treatment (ALTZ; anti-CD52). Thyroid eye disease (TED) can also develop, and rituximab (RTX; anti-CD20) is a suitable treatment.

**Case presentation:**

A 37-year-old woman with MS developed steroid-resistant active moderate-to-severe TED 3 years after ALTZ, that successfully responded to a single 500 mg dose of i.v. RTX. Before RTX peripheral B-cells were low, and were totally depleted immediately after therapy. Follow-up analysis 4 years post ALTZ and 1 year post RTX showed persistent depletion of B cells, and reduction of T regulatory cells in both peripheral blood and thyroid tissue obtained at thyroidectomy.

**Conclusion:**

RTX therapy successfully inactivated TED in a patient with low B-cell count derived from previous ALTZ treatment. B-cell depletion in both thyroid and peripheral blood was still present 1 year after RTX, indicating a likely cumulative effect of both treatments.

## Established facts

- Immunotherapy has become a mainstay in the management of autoimmune diseases.
- Combined therapy with different immunosuppressants has been studied poorly in autoimmune thyroid disorders.

## Novel insights

- Ours is the first description of the immune cell profiling derived from the consecutive administration of alemtuzumab for multiple sclerosis and rituximab for thyroid eye disease.
- B-cell depletion induced by rituximab is an effective treatment for thyroid eye disease, also in case of persistent reduced B-cell counts due to previous immunotherapy with alemtuzumab.

## Introduction

The humanized antibody anti-CD52+, alemtuzumab (ALTZ), has been approved since 2014 for the treatment of relapsing–remitting multiple sclerosis (MS). It causes panlymphopenia followed by lymphocyte repopulation, thus classified as an immune reconstitution therapy (IRT) (1, 2). After ALTZ, 34–41% of patients with MS develop thyroid autoimmunity, in 63–65% of whom characterized by Graves’ hyperthyroidism, caused by autoantibodies stimulating the thyrotropin receptor (TRAb) (3). However, TRAb that developed following ALTZ may also exert a blocking activity on the thyrotropin receptor, causing hypothyroidism, or switching thyroid function between hyper- and hypothyroidism if mixed with stimulating TRAb (4, 5, 6). Thyroid eye disease (TED) is an inflammatory disorder of the orbital tissues that occurs in 30–50% of patients with Graves’ disease (GD); moderate-to-severe forms account for 5–6% of cases (7, 8). TED has been reported in 13% of ALTZ-induced GD (4), although these figures might be underestimated. Few cases of ALTZ-induced TED have been reported until now, and their clinical characteristics were not consistent in terms of disease activity and severity, and response to disparate modalities of treatment (9, 10, 11).

The chimeric murine/human monoclonal antibody anti CD20+ rituximab (RTX) has been proposed as second-line therapy for active moderate-to-severe TED since 2016 (8, 12), and its efficacy has been compared with high-dose intravenous glucocorticoids (IVGC), which are generally used as first-line therapy (13). In addition to RTX, other alternative second-line treatments include an association of steroids with mycophenolate, cyclosporine, or azathioprine, orbital radiotherapy, tocilizumab, and teprotumumab (8, 14). In particular, RTX and tocilizumab are monoclonal antibodies that have been studied in steroid-resistant TED, and tocilizumab has been used successfully in ALTZ-induced TED (11).

To our knowledge, this is the first reported patient with ALTZ-induced TED resistant to high doses of IVGC, successfully treated with a single low dose of RTX. In this report, we describe the clinical response to treatment in this patient and provide data about the effects of consecutively administered ALTZ and RTX treatments on the main immune cell subsets.

## Materials and methods

### Clinical evaluation

The patient was referred to the combined thyroid-eye clinic of our TED Centre at the Endocrinology Department of Fondazione IRCCS Ca’ Granda Ospedale Maggiore Policlinico (Milan, Italy). TED activity and severity were defined according to the EUGOGO Guidelines (8) and the Consensus Statement by the American Thyroid Association (ATA) and the European Thyroid Association (ETA) (14). In particular, active TED was defined as the presence of a clinical activity score (CAS) above three points out of seven. We collected the patient’s clinical history, recorded, and monitored serum thyroid hormones and TRAb concentrations throughout the study period.

### TRAb assays

Automated TRAb was measured by Immulite 2000/2000 XPi TSI (Siemens, Erlangen, Germany) and considered negative if <0.55 KIU/L.

## Lymphocyte analysis

### Absolute lymphocyte counts

The absolute lymphocyte counts (number of cells/µL) were calculated at several time points on fresh blood using fluorescent microspheres (Beckman Coulter Life Science, Brea, CA, USA) and labeling cells with the following surface markers: CD45 (Clone 2D1; BioLegend, San Diego, CA, USA), CD3 (Clone OKT3; BioLegend), CD4 (Clone RPA-T4; BioLegend), CD8 (Clone RPA-T8; BD Pharmingen, Franklin Lakes, NJ, USA), and CD19 (1D3; ImmunoTools, Frieosythe, Germany). Data were acquired using a FACSCANTO flow cytometer (BD Biosciences).

### Immunophenotyping

At the time of thyroidectomy, immunophenotyping was performed on paired intrathyroidal and peripheral blood lymphocytes. Thyroid specimens were minced and digested with collagenase (1.0 mg/mL; Roche Molecular Biochemicals, Mannheim, Germany) for 1 h at 37°C. Thyroid-derived infiltrating mononuclear cells (TMCs) and peripheral blood-derived mononuclear cells (PBMCs) were isolated by Ficoll-Hypaque centrifugation and resuspended in complete RPMI 1640 medium supplemented with 10% fetal bovine serum. Isolated PBMCs and TMCs were stained using the following anti-human antibodies: CD45 (Clone HI30; BD Biosciences), CD4 (Clone SK3; BD Horizon), CD3 (Clone UCHT1; BD Horizon), CD19 (Clone UCHT1; BD Horizon), IL-7R (Clone HIL-MR-721; BD Horizon); CD25 (Clone M-A251; BD Biosciences), and Live/Dead-Aqua (L34957; Thermo Fisher). Data were acquired using a FACSymphony flow cytometer (BD Biosciences) and analyzed with FlowJo v10 software (FlowJo, LLC: BD Biosciences).

### Controls

Data from this patient were compared to those from three healthy donors (HD) from the transfusion center, all females with a mean age 28 years (PBMC only), and four patients with spontaneous GD (of whom two also had TED), two males and two females of mean age 37 years, undergoing thyroidectomy as per clinical indication (PBMCs + TMCs). Both HD and GD controls were also recruited from the Fondazione IRCCS Ca’ Granda Ospedale Maggiore Policlinico (Milan, Italy).

## Case report

A 37-year-old woman was diagnosed with relapsing–remitting MS and treated initially with natalizumab, a humanized IgG4 monoclonal antibody that selectively inhibits adhesion molecules, preventing leukocyte adhesion to endothelial cells (15). Subsequently, she was treated with fingolimod, a sphingosine-1-phosphate receptor modulator that prevents the egress of cells from secondary lymphoid tissues (16). IVGC were administered during exacerbations. Seven years after the initial diagnosis, due to increased MS activity, i.v. ALTZ was administered in two courses: 12 mg/day for five consecutive days, followed 1 year later by another cycle of 12 mg/day for 3 consecutive days.

**Figure 1:**
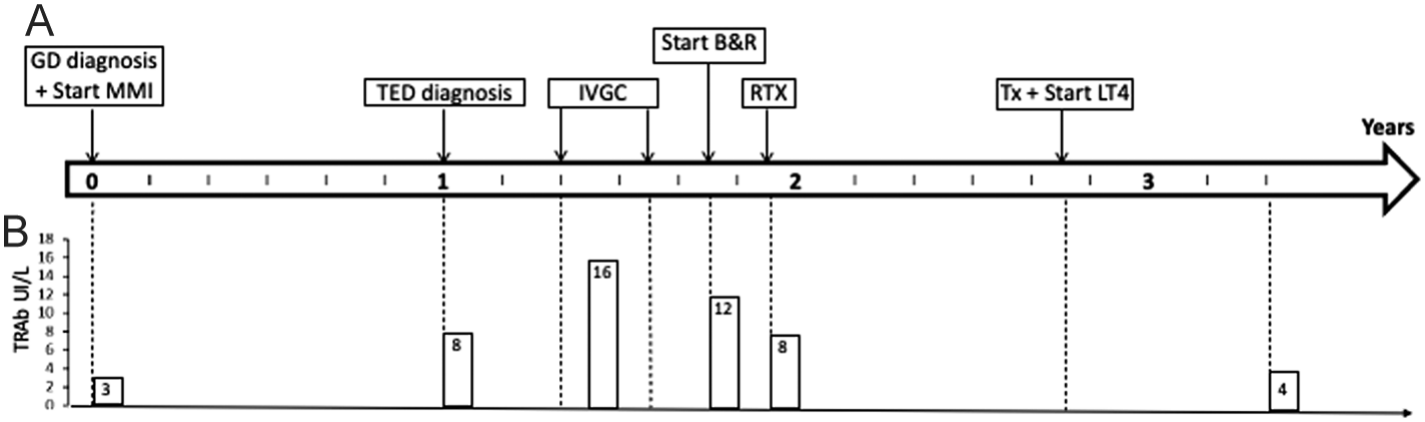
Longitudinal evolution. Timeline representing our patient’s clinical history (A), and serum concentration of autoantibodies stimulating the thyrotropin receptor (TRAb; B), from the diagnosis of Graves’ disease (GD), to the end of follow-up. B&R, block and replace (treatment with concomitant methimazole and levothyroxine); IVGC, intravenous glucocorticoids (4.5 g total cumulative dose of methylprednisolone administered weekly); LT4, levothyroxine; MMI, methimazole; RTX, rituximab (single 500 mg intravenous dose); TED, thyroid eye disease; Tx, thyroidectomy; Y, years.

After about 2 years from the last dose of ALTZ, the patient developed hyperthyroidism with detectable serum TRAb. She was an ex-smoker and had no family history of thyroid disease. Her thyroid scintigraphy scan showed diffusely increased thyroid technetium-99m uptake, and thyroid ultrasound revealed typical features of GD; thus, she was put on methimazole therapy. One year after the onset of GD, she developed progressive moderate-to-severe active TED, and was treated with weekly IVGC: methylprednisolone 4.5 g total cumulative dose (Fig. 1).

Despite therapy, her TED remained active. One month after the end of steroid treatment, the patient was referred to our TED Centre for further therapeutic management and presented the following characteristics: CAS 4, proptosis right/left eye 27/26 mm, intermittent diplopia, and lid retraction right/left eye 14/15 mm. Due to frequent fluctuations in thyroid function on methimazole treatment only, she was promptly commenced on the block and replace therapy with methimazole and levothyroxine, achieving good control of her hyperthyroidism. One month later, the CAS increased to 8, and she presented with proptosis 27.5/28 mm, intermittent diplopia, and lid retraction 10/12 mm. She was then treated with 500 mg i.v. RTX, which was well tolerated. After 1month, her TED was inactive, presenting a CAS of 3, proptosis 26/25 mm, constant diplopia, and lid retraction 10/12 mm. Two months after treatment, her CAS dropped to 1, while her proptosis (26/25 mm) and diplopia had remained stable; her lid retraction was slightly improved (9/10 mm). Ten months after RTX treatment, she underwent total thyroidectomy and achieved stable euthyroidism on levothyroxine replacement therapy. After only one dose of RTX treatment, TED remained persistently inactive and stable (CAS 0; proptosis 25/23.5 mm, constant diplopia), with further improvement of lid retraction (7/9 mm) up to 18 months of follow-up. The patient did not develop any infections or other complications secondary to the combination of immunomodulating therapies. Figure 1 summarizes the patient’s clinical evolution.

## Results

### Lymphocyte analysis

The patient's pre-treatment total lymphocyte count was normal: 1.37 × 10^9^/L (normal value (NV): 1.1–4.80 × 10^9^/L). Three days after the first ALTZ course, it dropped to 0.03 × 10^9^/L, with total depletion of CD19+ B cells (0%; NV: 7–21%). Four years later, before RTX, the patient’s peripheral CD19+ B-cell count was still persistently low (6.6%). The total lymphocyte count was also low (1.06 × 10^9^/L) and dropped to 0.32 × 10^9^/L 2 h after RTX infusion.

**Figure 2:**
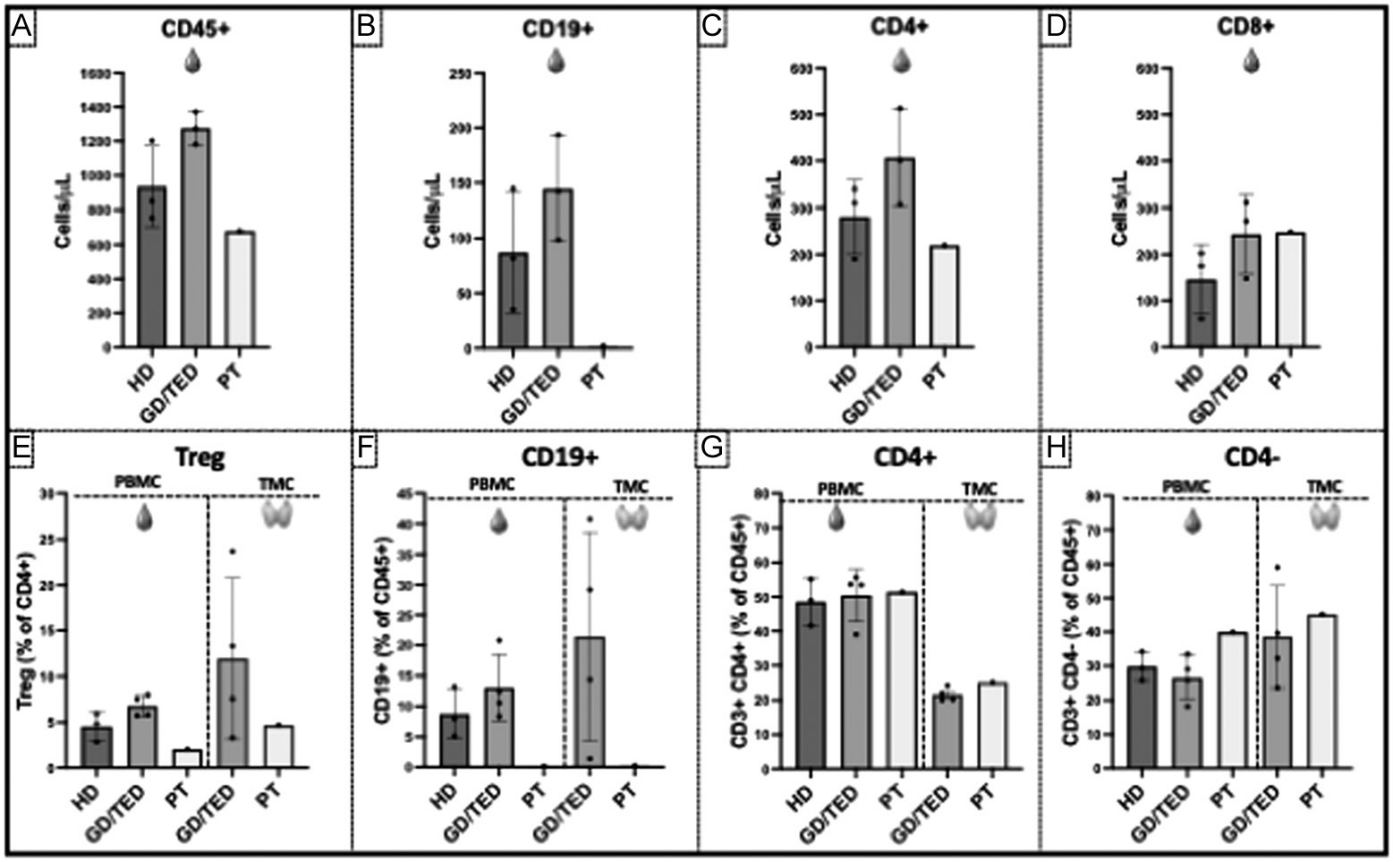
Immunophenotyping by flow cytometry. Lymphocyte analysis was conducted in our patient at the time of thyroidectomy (PT), three healthy donors (HD; *n* = 3), and four patients with spontaneous Graves’ disease, of whom two also had thyroid eye disease (GD/TED; *n* = 4). Upper bar plots show the absolute lymphocyte counts of CD45+ (A), CD19+ B (B), CD3+CD4+ (C), and CD3+CD8+ T (D) cells obtained from peripheral blood-derived mononuclear cells (PBMCs). Bottom bar plots represent the frequencies of regulatory (Treg; CD45^+^CD3^+^CD4^+^IL7R^low/-^CD25^+^) T cells on total CD4+ T cells (E), CD45^+^CD19^+^ B cells on total CD45+ cells (F), CD45^+^CD3^+^CD4^+^ Helper) T cells on total CD45+ T cells (G), and CD45^+^CD3^+^CD4^-^ Cytotoxic T cells on total CD45+ T cells (H) obtained from paired PBMC and thyroid-infiltrating mononuclear cells (TMCs).

Figure 2 shows the immunophenotyping analysis of paired blood- and thyroid-derived lymphocytes obtained at the time of thyroidectomy, 10 months after RTX, compared with the pattern observed in three healthy donors and four patients with spontaneous GD and TED. Both patient’s blood and thyroid tissue showed persistent B-cell depletion, as well as a reduction of CD4^+^IL7R^low/-^CD25^+^ T regulatory cells (Treg). The overall CD3+ T-cell count, as well as the frequencies of helper (CD4+) and cytotoxic (CD4-) T cells, did not differ between groups, neither in the peripheral blood nor in the thyroid gland.

## Discussion

To our knowledge, this is the first report of successful RTX immunotherapy in a patient with steroid-resistant TED developed after IRT for relapsing-remitting MS. The rapid inactivation of TED in our patient following B-cell depletion provides additional confirmation that RTX is an effective drug also in T-cell-mediated diseases, such as MS and TED (13, 17). In case of TED, a single 500 mg dose of RTX has been shown to inactivate the disease as effectively as a 1000 mg dose administered twice with a 2-week interval (13); therefore, the former dose is preferred in the majority of TED patients (18). Indeed, B cells may play pathogenic roles besides their activity of antibody-producing cells. It is plausible that their depletion likely interrupts the early antigen presentation activity, which sustains the autoimmune process (19, 20). As seen in other studies (21, 22), RTX did not seem to affect the levels of circulating TRAb, since it does not reduce plasma cells (23, 24). In this patient, TRAb titers had already started to decrease after achieving good control of hyperthyroidism (23, 24) and never became negative, even 1 year after therapy with RTX. Hyperthyroidism did not undergo remission, and the patient was referred for thyroidectomy.

The pathogenic mechanisms underlying the induction of autoimmune disorders by IRT, particularly ALTZ, remain controversial but may relate to the complex kinetics of lymphocyte repopulation. ALTZ causes a rapid panlymphocyte depletion followed by a multistep immune reconstitution process. Total B-cell numbers quickly recover to baseline values, or even exceed them, within 3–6 months (1, 25, 26, 27, 28). Other studies observed a slower median recovery time of 8.4 months; only a few patients reached a final B-cell count below the baseline values (29). On the other hand, after ALTZ T lymphocytes repopulate much slower, driven by homeostatic proliferation of survived cells. T-cell depletion persists for several years, and the T-cell count usually does not recover to baseline numbers, especially the CD4+ subset (27, 29, 30, 31). During the first months after ALTZ treatment, there is an exaggerated repopulation of naïve B cells associated with T-cell scarcity, possibly leading to a lack of regulation (32). However, secondary autoimmunity usually develops 2–3 years after IRT, and until now the reason for such late-onset has not been explained (28). T-cell reconstitution by homeostatic proliferation is characterized by the relative expansion of the T regulatory subtype with enhanced suppressing activity, which may persist up to 48 months (28, 31, 33, 34, 35). Other studies have observed a reduction in the Th1 and Th17 cell subsets, likely as a consequence of the enhanced activity of Treg cells, which could explain the persistent clinical remission of MS (28, 33, 34).

RTX induces an immediate depletion of B cells but not plasma cells. The immune cell repopulation kinetics depend on the dose and treatment schemes used, which vary among different diseases. When RTX is administered as a treatment for MS, B cells return in the peripheral blood in about 20–24 weeks, depending on the dose and the schedule of administration, although they may take up to 12 months to normalize (36, 37). RTX may also indirectly induce T-cell depletion in both PBMC and cerebrospinal fluid of patients with MS, which can persist for several weeks, and it has been itself associated with treatment efficacy (36, 38, 39, 40). Other authors have not reported reduced T-cell counts after RTX therapy for MS but rather a profound change of their subsets (41). Scarce and conflicting data are available about Treg kinetics following RTX. Patients with follicular lymphoma showed a Treg reduction after RTX treatment (42), while another study, where RTX was used to treat pemphigus vulgaris, did not show a variation in the Treg population after treatment (43). Studies about RTX administration for lupus nephritis, non-Hodgkin lymphoma, and idiopathic thrombocytopenic purpura, instead showed an increase in Treg post treatment (44, 45, 46, 47). The reasons for such discrepancies can depend on the heterogeneous diseases evaluated and the different markers used to identify Treg cells. In our study, we used CD45^+^CD3^+^CD4^+^IL7R^low/-^CD25^+^ as a validated strategy to select Tregs using surface-only markers (48). To our knowledge, only two studies evaluated the lymphocyte repopulation kinetics following low doses of RTX administered for TED treatment. The B cells began repopulating in 4–12 weeks and reached the 53–70% of basal levels in 40–76 weeks, depending on the different doses and schedules of RTX administration, while no T-cell depletion was observed (18, 21).

In our patient, the peripheral B-cell counts remained as low as 6.6% (NV: 7–21%) at 4 years after ALTZ, as occasionally reported (29) elsewhere, and after RTX became undetectable for the following 10 months. This is likely the result of a cumulative effect of ALTZ and RTX, further hindering the B-cell repopulation (36). Despite having persistently low B cells, RTX therapy effectively inactivated TED, which was unexpected and previously unreported. Despite the relative decrease in the Treg population after ALTX and RTX sequential treatments, both her TED and MS did not relapse. In addition, the patient did not develop post-IRT infections, as described for the majority of patients treated with ALTZ (33) and also RTX (17).

In conclusion, the use of targeted monoclonal antibodies as immunotherapy for TED proves to be effective, safe, and can help better understand the immunological mechanisms underlying the disease. In recent years, the role of B-cell antigen presentation has gained importance in the pathogenic mechanisms of TED, based also on the evidence obtained with RTX therapy. This case raises several open questions that need dedicated larger studies to be addressed. First, despite this patient presenting prolonged partially depleted B-cell counts following ALTZ, she developed severe GD with active moderate-to-severe TED. Secondly, despite the pre-treatment decrease in B-cell numbers, RTX was highly effective in inactivating her TED. Thirdly, after 18 months of follow-up, both her MS and TED remained in remission despite a relative decrease of Treg frequencies. The study of combined immunological effects deriving from the use of different immunotherapies is important to define a correct safety and efficacy profile.

## Declaration of interest

MS is a consultant for IBSA, ArgenX, and Immagene and has received speaker’s fees from IBSA. The Institution Fondazione IRCCS Ca’ Granda Ospedale Maggiore Policlinico Milan has received research funding from Roche, GSK, Viridian, Sling Therapeutics, Immunovant, and Horizon Pharma. The other authors have no competing interests.

## Funding

This study was supported by the Ricerca Corrente Funds from the Italian Ministry of Healthhttp://dx.doi.org/10.13039/100009647 to Fondazione IRCCS Ca’ Granda Ospedale Maggiore Policlinico Milan, the Rita Levi Montalcini Program for Young Researchers – Italian Ministry of University and Research (MUR) to IM, the Early Career Grant from the Society for Endocrinologyhttp://dx.doi.org/10.13039/501100000382 (SfE) to IM, and the European Thyroid Association (ETA) Research Grant to IM.

## Statement of ethics

This study was approved by the ethical committee of Milano Area 2 (permission 0004946 69_2020bis 7/2/2020 ID 1333), and the patient agreed to participate in the study.

## Author contribution statement

IM raised funds, took charge of patient management, wrote the article, contributed to study conception and design. SM and JG performed the immunological analysis, reviewed, and edited the article. MA and LPo collected patient’s data, did the literature search, contributed to figures, reviewed and edited the article. NC, EI, LPi, JM, and CM took charge of patient management, collected patient’s data, reviewed and edited the manuscript. MS took charge of patient management, conceived the study, gave intellectual input into design, reviewed and edited the manuscript.

## Acknowledgements

The authors acknowledge the support of the APC central fund of the university of Milan.
